# Identifying Priorities for the Department of Veterans Affairs Strategic Plan: A Rapid Multi‐Method Evaluation

**DOI:** 10.1002/lrh2.70068

**Published:** 2026-02-12

**Authors:** John P. Donnelly, C. Ann Vitous, Kaylee W. Burgan, Gezan M. Yahya, Jessica L. Johnson, Jessica A. McDonald, Nicholas W. Bowersox, Linda M. Kawentel

**Affiliations:** ^1^ VA QUERI Center for Evaluation and Implementation Resources Ann Arbor Michigan USA; ^2^ VA HSR Center for Clinical Management Research Ann Arbor Michigan USA; ^3^ Department of Learning Health Sciences University of Michigan Ann Arbor Michigan USA; ^4^ School of Information University of Michigan Ann Arbor Michigan USA; ^5^ Center for Health Outcomes and Policy University of Michigan Ann Arbor Michigan USA; ^6^ Department of Family and Community Medicine University of Alabama at Birmingham Birmingham Alabama USA; ^7^ Department of Psychiatry University of Michigan Ann Arbor Michigan USA

**Keywords:** rapid evaluation methods, strategic planning, veteran priorities

## Abstract

**Background:**

Understanding the priorities of patients and leaders can help organizations prepare for the future. This evaluation sought to generate actionable data on priorities in support of the Department of Veterans Affairs (VA) strategic plan, with priorities identified within the literature as well as among Veterans and VA leaders.

**Methods:**

A rapid qualitative evaluation was conducted, including a document analysis based on published information, focus groups with Veterans associated with research centers, and semi‐structured interviews with VA leaders. All data were analyzed using rapid qualitative methods, resulting in comprehensive templated descriptions of topics discussed and representative quotes. Summative content analysis was used to report frequencies and identify the most frequently mentioned priorities.

**Results:**

A total of 170 documents (e.g., academic/research articles, government publications) were analyzed. Seventeen Veteran focus groups (92 participants) and 26 semi‐structured interviews with VA leaders (representing 19 program offices) were conducted. There were differences in the most frequently mentioned priorities across sources, with none ranked in the top 5 for all. However, “Access and Continuity of Care,” “Health Benefits,” “Special Groups,” and “Workforce” were among the top 10 mentioned within all sources. Among these, “Workforce” was most mentioned within the document analysis, whereas “Access and Continuity of Care” was most mentioned in focus groups. Both “Health Benefits” and “Access and Continuity of Care” were most mentioned during leadership interviews.

**Conclusions:**

Our findings suggest shared priorities to emphasize as VA looks to the future: improving care access, efficient and effective benefits processing, ensuring the needs of special Veteran groups are met, and sustaining an adequate workforce. However, further work should focus on understanding how Veterans and staff engage with the system to guide the investment of limited organizational resources. This work serves as an example of how rapid evaluation could be applied for strategic planning in other settings.

## 1 Introduction

Strategic planning is essential for organizations to navigate challenges, prioritize resources, and prepare for the future. An important product of the planning process is the strategic plan, which defines an organization's goals and the methods for achieving those goals. This is especially important for large healthcare organizations, as strategic planning provides a systematic, rational, and integrative approach to managing the most relevant, feasible, and necessary transformations for the future [1]. Within the Department of Veterans Affairs (VA), the strategic plan is updated every 4 years.

An important first step in the VA Strategic Plan development process is to understand the current state of Veteran care and systematically identify priority areas to “help [VA] execute [their] mission of serving Veterans, their families, caregivers and survivors as well as they have served [VA]” [2]. It can be challenging to ensure that the interests of different parties within a health system are adequately represented, and it is possible that priorities could be in tension with each other (e.g., health care cost containment and expansion of access to care). This highlights the need for a broad approach that includes opportunities for interested groups to voice their perspective, with active participation from clinicians, administrators, patients, and caregivers [3, 4]. Due to the changing nature of health care, time constraints, and organizational capacity, there is a need for pragmatic data collection approaches that allow for rapid, evidence‐based decision‐making [5, 6]. Rapid evaluation offers a time‐sensitive approach to understand priorities and focus areas for development [6, 7, 8, 9]. There has been limited scholarship on the use of rapid evaluation methods to inform strategic planning efforts within large integrated health systems [6, 8].

Within VA, the Office of Enterprise Integration (OEI) has led efforts to create and update the VA Strategic Plan, a guiding document that spans VA's three administrations—the Veterans Health Administration (VHA), Veterans Benefit Administration (VBA), and National Cemetery Administration (NCA). To support the creation of the FY2026–FY2030 VA Strategic Plan, OEI requested that the VA Quality Enhancement Research Initiative (QUERI) Center for Evaluation and Implementation Resources (CEIR) generate data on the current state of VA priorities. In this context, a rapid evaluation was conducted to identify VA priority areas as of 2024 using multiple methods: a review and analysis of existing literature, focus groups with Veterans, and semi‐structured qualitative interviews with VA leaders.

## 2 Research Interest

Identifying priorities is an important early step in strategic planning, and it is unclear to what extent key interested groups share priorities for the future of VA. This evaluation assessed VA priorities using qualitative methods and compared top priorities across the published literature, Veterans, and VA leaders.

## 3 Methods

### 3.1 Study Design

This work included a several step process to identify VA organizational priorities across interested groups: (1) a document analysis to identify priorities from relevant literature; (2) focus group sessions with VHA Health Systems Research (HSR) Center of Innovation (COIN) Veteran Engagement Panels (VEPs) to identify priorities for Veterans; and (3) key informant interviews with VA leaders, including members of the VHA Evidence‐based Policy Subcommittee (EBPS). VEPs are made up of Veteran representatives who offer their feedback on VA projects and are consulted by research teams, providing an existing infrastructure to gain input from Veterans in a rapid manner that is well‐suited to the needs of this work. The EBPS serves as the principal point of contact for the VHA under Secretary for Health to ensure fulfillment of core requirements of the Evidence Act, which requires federal agencies to submit systematic plans for identifying and addressing policy questions.

Data collection took place over a 4‐month period (December 2023 through April 2024). According to the regulations outlined in Program Guide 1200.21, this evaluation was designated as a non‐research quality improvement project and was exempt from Institutional Review Board oversight [10].

### 3.2 Document Review

A document analysis was conducted that involved reviewing reports, publications, and news articles [11]. Targeted searches were conducted to identify documents from the following sources: academic/research databases (e.g., JSTOR, PubMed, and Veteran‐focused journals not indexed in these repositories); government websites; nationally syndicated newspaper websites with searchable databases; Veteran advocacy and assistance group websites; research organization websites; and VA staff group websites. Appendix SS1 outlines inclusion criteria based on relevance to priority areas, national scope, pragmatic implications (clear indication of problem and recommendation), and publication between 2016 and 2024. Appendix S2 includes all terms used to perform database searches. PDFs of all documents for full‐text review were uploaded into MAXQDA (VERBI Software, Berlin, DE).

### 3.3 Veteran Focus Groups

Veteran perspectives were assessed by conducting focus groups with VEP members, with 23 COIN‐affiliated VEPs contacted for scheduling [12]. A semi‐structured focus group interview guide (Appendix S3) was developed and administered via virtual video teleconferencing meetings (Zoom for Government, Microsoft Teams, or Webex). Each focus group session was led by a moderator trained in qualitative research methods, accompanied by two note‐takers. Note takers took detailed notes, capturing verbatim quotes when possible (due to operational partner preferences, sessions were not recorded). Each focus group lasted approximately 60 min and began with an overview and explanation of the study's purpose. All participants provided verbal consent.

### 3.4 Semi‐Structured Interviews With VA Leaders

Interviews were conducted with members of the EBPS and other VA leaders recommended via snowball sampling [13]. Participants were recruited via email for semi‐structured qualitative interviews, using an interview guide (Appendix S3). All participants gave verbal consent and interviews were conducted by project staff with qualitative interviewing experience via Zoom for Government or Microsoft Teams. All interviews took no more than 60 min and were conducted in a similar manner as the VEP focus groups. At the end of each interview, participants were asked to identify any additional VA leaders they felt could contribute meaningful insights.

### 3.5 Analysis

For the document analysis, a hybrid inductive/deductive approach was used to establish a codebook for priorities VA should emphasize in the future (Appendix S4) [14]. Team members independently reviewed three documents and then met to reach consensus on initial codes. Documents were then assigned and independently coded. The project leads merged the files of coded documents into a single primary file and conducted a descriptive content analysis [15]. Once priority areas were determined, reports were generated to identify coded excerpts and assemble analytic matrices with summaries of identified problems and recommendations. Lastly, queries were run to assess the frequency of individual codes applied to unique documents.

Rapid qualitative approaches were used to analyze focus group and interview data. Rapid qualitative analysis is commonly used in VA health services research to obtain actionable, targeted data on a shorter timeline than traditional qualitative methods while still following accepted scientific processes and rigor [16]. Specifically, two staff members took notes during the sessions using a template with domains aligned with focus group or interview questions. A third team member then consolidated the two sets of notes, edited them, and transferred the content into a matrix. Each row in the matrix corresponded to a given focus group or interview, while each column referred to a unique domain from the guide. Matrix columns were then analyzed by multiple team members for similarities, differences, and trends. Discrepancies between analysts were resolved by consensus. As an additional step in the analytical process, the matrix domain “Veteran and VA Needs” was analyzed using the codebook developed for the document review (Appendix S4).

Summative content analysis was used to calculate frequencies, compare distributions of commonly identified priorities, and report the top priorities within each group [17]. The aim of this analysis was to explore differences in reported priorities across data collection methods, but it is important to clarify that differences in the frequency of mentions would not necessarily reflect relative importance.

## 4 Results

### 4.1 Document Review and Content Analysis

Of the 296 identified documents, 170 (57.4%) were included in the document analysis (Appendix S5). Among included documents, 53 (31.2%) were from academic/research databases, 44 (25.9%) were from government sources, 33 (19.4%) were articles from nationally syndicated newspaper websites, 21 (12.4%) were from Veterans advocacy and assistance groups, 12 (7.1%) were from research organizations, and 7 (4.1%) were from sources relevant for VA staff.

Table 1 provides frequency counts of the most commonly referenced priority areas by source. Appendix S6 provides the frequency counts for all priority areas. Table 2 shows the specific topics discussed by priority area, as identified by document content analysis. The documents focused on a broad range of Veteran needs across different population groups and settings.

**TABLE 1:** Frequency counts of top 10 priority areas by source in document analysis.

Priority area	Document source						
	*N* (column %)						
	Academic databases (*N* = 104)	Government (*N* = 92)	News sources (*N* = 40)	Research organizations (*N* = 41)	VA staff (*N* = 8)	Veterans advocacy and assistance groups (*N* = 87)	Row total
Homelessness^a^	20 (19.2%)	9 (9.8%)	5 (12.5%)	4 (9.8%)	0 (0%)	3 (3.4%)	41 (13.9%)
Workforce^a^	2 (1.9%)	13 (14.1%)	3 (7.5%)	3 (7.3%)	5 (62.5%)	8 (9.2%)	34 (11.5%)
Data management	3 (2.9%)	17 (18.5%)	3 (7.5%)	3 (7.3%)	0 (0%)	6 (6.9%)	32 (10.8%)
Veteran belonging	17 (16.3%)	3 (3.3%)	1 (2.5%)	3 (7.3%)	0 (0%)	7 (8.0%)	31 (10.5%)
Women's health	12 (11.5%)	2 (2.2%)	2 (5.0%)	4 (9.8%)	1 (12.5%)	8 (9.2%)	29 (9.8%)
Special groups	10 (9.6%)	10 (10.9%)	2 (5.0%)	1 (2.4%)	0 (0%)	5 (5.7%)	28 (9.5%)
Access and continuity of care^a^	4 (3.8%)	7 (7.6%)	1 (2.5%)	5 (12.2%)	1 (12.5%)	8 (9.2%)	26 (8.8%)
Health benefits	4 (3.8%)	4 (4.3%)	1 (2.5%)	7 (17.1%)	0 (0%)	9 (10.3%)	25 (8.4%)
Education, training, employment	1 (1.0%)	6 (6.5%)	3 (7.5%)	5 (12.2%)	0 (0%)	10 (11.5%)	25 (8.4%)
Conditions and treatments	8 (7.7%)	5 (5.4%)	6 (15.0%)	0 (0%)	0 (0%)	6 (6.9%)	25 (8.4%)

^a^ FY2024‐25 VA agency priority goals.

**TABLE 2:** Topics discussed by priority area in document analysis.

Priority area	Topics discussed
Homelessness^a^	Housing (e.g., transitional housing, rental assistance) Outreach efforts Food insecurity Variation in needs based on subgroup (e.g., sex, race) Unmet health needs (e.g., dental care, mental health) Use/preference for community care
Workforce^a^	Understaffing (e.g., rural areas, providers, nurses) Workload burdens Lack of knowledgeable providers High turnover rates (e.g., leadership, providers) Long onboarding and hiring processes Need for better human capital management
Data management	Lack of data on quality‐of‐care coordination among the private sector, VA clinicians, and outcomes of Veterans Inefficiencies of systems Protecting the privacy of sensitive information
Veteran belonging	Need for reform that ensures Veterans feel welcomed when engaging with the system as a whole Need to address increasingly diverse population of Veterans
Women's health	Unmet needs of mental health issues that are specific to women Ensuring that women Veterans have access to sex‐specific care Sexual harassment of women Veterans at VA
Special groups	Unmet needs of rural Veterans (e.g., medication, care, providers) Unmet needs of Veterans with physical and mental disabilities Unmet needs of aging Veterans (> 65) Unmet needs of other Veterans
Access and continuity of care^a^	Proximity/geographic alignment of care Caps on service quantity Access to services is distributed inconsistently or and/or inequitably Unmet transportation needs Variation in timeliness and wait times across VA's system
Health benefits	Lack of awareness of benefits and how to access Uncovered costs Eligibility criteria Lack of effective processes for obtaining information needed to resolve pending applications
Education, training, employment	Underemployment Predatory recruiting practices of some for‐profit colleges Lack of relevant data to inform employment outcomes Lack of understanding on resources, benefits, and/or processes Single parents need additional guidance on career paths
Conditions and treatments	Need for increased mental health and substance use counseling Need for alternative therapies for pain management Need for increased attention to needs of subgroups Data limitations on conditions and treatments

^a^ FY2024‐25 VA agency priority goals.

### 4.2 VEP Focus Groups

Of the 23 VEPs contacted, 16 focus groups were completed, while an additional VEP sent written responses to the questions (participation rate 74%). Focus group sizes ranged from 2 to 10 participants (median, 5.5 participants), and included a total of 92 Veterans. Table 3 summarizes the top 10 priority areas, ordered by most to least cited, and presents specific unmet needs and challenges discussed by the Veterans. The quotes and descriptions provided below help to contextualize these findings for each priority.

**TABLE 3:** Unmet Veteran needs and challenges by priority area in VEP focus groups.

Priority area	Needs and challenges discussed
Access and continuity of care	Long wait times Need to expand operational hours (e.g., weekends) Contact/coordination between Primary Care and Specialists Lack of access to certain services (e.g., end‐of‐life care, primary care, chiropractic care, emergency care) Insufficient communication and secure messaging with care team Insufficient access to preventative care Insufficient access to mental health care
Customer relations	Internal stigma regarding purpose of VA (e.g., perception that VA is for older Veterans) Lack of education and outreach to all Veterans on benefits and services available and how to access Treatment to Veterans (e.g., lack of politeness by VA staff)
Quality and consistency of care	Stigma about quality of care (e.g., reliability, quality of services, previous negative experiences) Lack of accountability (e.g., providers) Lack of consistency in quality of care and services offered across VAs Insufficient training for providers and staff (e.g., crisis training in emergency departments) Treatment of symptom rather than cause
Veteran belonging	Diversity of providers (e.g., age, sex, race) Lack of congruence between provider and Veteran Evaluation for benefits Access to care and services available for Veteran subgroups (e.g., women, era of service)
Young veterans	Assistance with transition into civilian life (e.g., employment) Underutilization of services Increased risk of SUD post deployment
Community care and privatization	Uncertainty about quality of care (e.g., “treatment mills”) Lack of access and long wait times to see private providers Delays in reimbursement to civilian providers Referrals to community care often unavailable
Civilian transition and outreach	Overreliance on non‐profits (e.g., during transition) Lack of education and outreach on benefits and services available
Health benefits	Reconsider eligibility based on level of service disability Need for additional coverage (e.g., dental, vision)
Workforce	Personnel shortages and retention issues (e.g., nurses, doctors) Efficiency of onboarding Workload burdens (e.g., nurses, doctors)
Other	Underutilization of VHA Integration between VBA, VHA, NCA Difficulty navigating bureaucracy Need to prioritize changes that are most valuable to Veterans (rather than hot topics) Need for more assistance and resources for trust and wills Need to standardize process for obtaining advanced directives

#### 4.2.1 Access and Continuity of Care/Customer Relations

The most frequently mentioned priority among the VEPs was access and continuity of care (e.g., increased communication between PCPs and specialty providers). VEP participants also reflected on the need to improve customer relations as the next most frequently mentioned priority, emphasizing the need to address factors such as stigmas, including the fear of taking away services from others who “need [them] more” (VEP1), and the belief that if you are healthy, VA services are not for you.

#### 4.2.2 Quality of Care

Another priority area mentioned by some participants was the lack of consistency and quality of care across the system (i.e., “You can go from one VA to another and it's like a 180‐degree difference in care” (VEP10)), as well as a need for providers to be more accountable.

#### 4.2.3 Veteran Belonging

Some participants also described Veteran belonging concerns, including a need for more congruence between providers and the Veterans they serve, the lack of adequate care for specific Veteran subgroups, and a need to improve how Veterans are evaluated for benefits. Additionally, some noted that many newer Veterans are not utilizing VA due to perceptions of VA being for older Veterans, with one participant stating, “It feels like I don't belong here and need to wait my time.” (VEP15).

#### 4.2.4 Community Care

Some participants reflected on unmet needs of community care, including concerns related to the quality of care provided by non‐VA providers, wait times, and the lengthy reimbursement process for claims from non‐VA providers (“I can't imagine how difficult it would be to a civilian provider trying to navigate the culture and paperwork and processes” (VEP15)). Other participants expressed concerns about long wait times and the difficulty in obtaining referrals. As one participant asserted, “I get care at the VA but would like to be able to see someone in the civilian world that can get me in to be seen quicker without all the rigmarole!” (VEP5).

#### 4.2.5 Civilian Transition

Some participants also described the need for increased attention toward civilian transition and outreach. As one participant explained, “Where in the country can I go that my job skills are transferable? Salary? How do I get in touch with these people and apply?” (VEP3). Although not a common sentiment, some asserted that there tends to be an overreliance on resources provided by non‐profit organizations.

#### 4.2.6 Health Benefits

Some participants mentioned the need to expand health benefits, with a particular focus on reconsidering eligibility and its relationship to service disability. As one participant asserted, “You shouldn't (need to) be deemed disabled to get support from the US military and VA” (VEP3). Others focused on the need for additional coverage (e.g., chiropractic care, dental, vision).

#### 4.2.7 Workforce

Some participants also reflected on workforce challenges, with a shortage of nurses and doctors of greatest concern to Veterans. Participants also cited concerns with provider retention and heavy workloads, discussing how these challenges could impact Veteran care.

#### 4.2.8 Other Priorities

Lastly, participants identified other priority areas. Several asserted the need for increased integration of VA administrations, while others stated that the VA should prioritize quality and access to care over budget and funding, or “hot topics.” Finally, some described a need to provide increased assistance with specific benefits or services (e.g., trusts and wills, advance directives).

### 4.3 Leadership Interviews

Of the 29 leaders, 26 agreed to participate in interviews (90% participation rate), representing 19 VA program offices. Table 4 summarizes the top 10 priority areas, listed in order from most cited to least. The quotes and descriptions provided below help to contextualize findings related to unmet needs and challenges, as identified by VA leaders.

**TABLE 4:** Unmet Veteran needs and challenges by priority area in leadership interviews.

Priority area	Needs and challenges discussed
Customer relations	Lack of clear, consistent communication with Veterans and their families regarding VA benefits, services, and resources Negative perceptions about VA and care received at VA Veterans' internalized stigma and pride as barriers to seeking care Need for targeted outreach to specific Veteran subgroups Need for increased visibility and transparency regarding claims processes, eligibility, and access to care
Access and continuity of care	Growing wait times and connection to services Managing access to mental health care Lack of technological innovation to improve access (e.g., telehealth, AI) Access to care for women Veterans Concerns about maintaining and expanding long‐term care access for aging Veterans
Health benefits	Fear of VA's capacity to meet growing and changing needs of Veterans Need to increase awareness of benefits and eligibility Need to expand benefits and services
Civilian transition and outreach	Veterans' struggle transitioning from active duty to civilian life Need to improve socialization skills after leaving active duty Lack of communication between DoD and VA Veterans' lack knowledge of available benefits upon leaving active duty Need for greater support to Veterans seeking employment after active duty
Community care and privatization	Challenges in predicting trends in community care usage Difficulty coordinating community care versus direct VA care Concerns about VA become a billing model for community care rather than a healthcare model for direct care Delays in reimbursement for providers
Special groups	Needs of aging Veterans will be different than the needs of younger Veterans Aging Veterans, particularly rural aging Veterans, will have more difficulty getting to medical appointments Women Veterans' discomfort with VA and receiving care at VA Need for improved outreach to homeless Veterans
Data management	Managing and transitioning the electronic health record (EHR) Reconciling and governing VA's data Need for better data on Veteran migration and demographics
Budget and funding	Limits on funding availability for services and benefits Inability to effectively steward resources for care Inability to pay for long‐term care in Veterans' preferred setting Difficulty coordinating and financing care between VA, Medicare, and Medicaid Concerns about financing virtual care moving forward
Workforce	Challenges growing and maintaining VA's workforce, particularly following PACT Act
Young veterans	Challenges engaging younger Veterans in VA care Fewer interactions and social connections with other Veterans

#### 4.3.1 Customer Relations

Participants identified the need to improve customer relations with Veterans as the most frequently mentioned priority area, with a particular emphasis on clear communication and a need to enhance trust and accountability in the eyes of Veterans.

#### 4.3.2 Access and Continuity of Care

Many participants also discussed challenges related to access and continuity of care. Concerns related to this area included growing wait times for establishing a Veteran service connection, a lack of clarity among Veterans about how to access their benefits, and uncertainty in terms of the health system's ability to respond to growing access needs in a timely manner.

#### 4.3.3 Health Benefits

Some participants also reported concerns regarding VA's existing health benefits, emphasizing the need for VA to help Veterans make “informed decisions” (VL7) on their care options, to help them navigate the system, and to grow to meet the expanding needs of Veterans and their families.

#### 4.3.4 Civilian Transition

When asked about Veteran challenges and needs more specifically, some participants discussed challenges related to service members' transition to civilian life following active duty and the lack of awareness of benefits and services among this group. This concern was linked to a need for improved collaboration between the VA and the Department of Defense (DoD) to support a seamless transition from military to civilian life.

#### 4.3.5 Community Care

Some participants also reflected on challenges resulting from the growing utilization of community care, such as difficulties with care coordination, VA shifting *to* “a billing model instead of a healthcare model” (EBPS3), and the idea that “VA has become a payer more than a provider” (VL1).

#### 4.3.6 Special Veteran Groups

Some participants also reported many needs regarding special groups of Veterans, most notably aging, women, and unhoused Veterans. Participants emphasized that aging Veterans' needs are different from those of younger Veterans and that VA will need to think carefully about caring for rural aging Veterans, who will face additional challenges as they “become more homebound and less mobile” (VL2).

#### 4.3.7 Data Management

Some participants also emphasized the need to improve data management, specifically the need to manage the electronic health record transition, determine proper data governance across administrations, and utilize the data resource to track Veterans' changing demographic and geographic patterns.

#### 4.3.8 Budget and Funding

Some participants also reported on budget and funding challenges within VA. Specifically, participants expressed concerns about VA's ability to effectively allocate resources given the growing utilization of community care, the ability to pay for long‐term care for Veterans, and challenges in coordinating finances across VA, Medicare, and Medicaid. Participants reported concerns about the limits Congress puts on VA's available budget, wondered how VA will balance this with the growing cost of services, and emphasized that *how* VA responds to these budget limitations is just as important as the limitations themselves.

#### 4.3.9 Workforce

Some participants noted challenges related to VA's workforce. Most notably, participants reported the lack of available providers, which will greatly impact VA's capacity and ability to meet access targets. Participants noted the need to appropriately allocate funds and focus on recruitment and retention to maintain a quality workforce and “keep the right people in the right positions” (VL8), as well as ensuring the whole health of staff to ensure better care delivery for Veterans.

#### 4.3.10 Other Priorities

Some participants also reflected on the needs of young Veterans. Most notably, participants reported challenges in engaging younger Veterans, as methods and preferences for contact differ for this population compared to previous generations.

### 4.4 Comparisons Across Data Collection Methods

Table 5 presents the top priority areas identified overall and stratified by data collection method. None of the identified priorities were ranked in the top 5 for all data collection methods. However, “Access and Continuity of Care,” “Health Benefits,” “Special Groups,” and “Workforce” were consistently discussed across all data collection methods (ranked in the top 10 for all). Among these priority areas, “Workforce” was most frequently addressed in the document analysis, whereas “Access and Continuity of Care” was most frequently discussed in the VEP focus groups. Both “Health Benefits” and “Access and Continuity of Care” were discussed frequently during leadership interviews.

**TABLE 5:** Top priorities discussed overall and by data collection method.

Priority area	Document analysis	VEP focus groups	Leadership interviews^a^	All
Access and continuity of care				X
Budget and funding				
Civilian transition and outreach				
Community care and privatization				
Conditions and treatments				
Customer relations				
Data management				
Veteran belonging				
Education, training, employment				
Health benefits				X
Homelessness				
Quality and consistency of care				
Special groups				X
Women's health				
Workforce				X
Young veterans				

^a^ Includes snowball sample interviews. Dark blue represents a priority area ranked in the top 5 by frequency. Light blue represents a priority area ranked in the top 10 but not the top 5 by frequency. X represents priority areas in the top 10 across all methods.

## 5 Discussion

Strategic planning can help to ensure the development of a shared vision within large governmental and healthcare organizations. We conducted a multi‐method rapid evaluation to identify VA priorities that should be emphasized within the next VA Strategic Plan. We identified an overall list of priorities, with four mentioned consistently across all data sources: “Access and Continuity of Care,” “Health Benefits,” “Special Groups,” and “Workforce.”

Our findings represent a first step in the strategic planning process. The process includes the following planned next steps: conducting surveys and interviews with Veterans and employees, performing a gap analysis, developing key questions, identifying themes and solutions, issuing summary documents, and developing a draft of the strategic plan. However, we can offer several recommendations based on our current findings related to specific strategies to address common priority areas. For “Access and Continuity of Care,” enhanced and transparent tracking of Veteran needs, along with refining quality measures related to care quality and access, could address many factors associated with this concern. In terms of the VA “Workforce,” this priority was mentioned most frequently in the document analysis, with less frequent mention among focus groups with Veterans or interviews with leaders. This suggests VA staff producing articles and other documents may be most aware of workforce needs. Potential recommendations include monitoring staffing levels, expediting hiring and onboarding processes, and adopting creative approaches to utilize employees in nontraditional ways, which could help improve facility performance and Veteran outcomes [18]. For VA “Health Benefits,” potential strategies could focus on streamlining the benefits process for specific groups and providing clear communication of Veteran benefits and opportunities. Lastly, for care for “Special Groups” of Veterans, key strategies would include additional attention, expanded service offerings, and enhanced screening that target these groups.

We were able to rapidly identify priorities using a multimodal approach that could be replicated in other organizations engaged in strategic planning. Our approach was unique in that we worked closely with multiple interested groups, including Veteran patients, to identify priority areas. For other organizations outside of VA, it is equally important that patients are included in planning processes. Such inclusion sends a strong signal that patients serve not just as data sources but also as active partners in helping to shape the future of health organizations. Although our approach included collecting feedback directly from Veteran patients, additional work is needed to fully integrate patient voices. Within VA, this could involve promoting patient engagement in broader planning efforts, such as encouraging Veteran and Veteran Service Organization participation in facility leadership meetings, establishing more Veterans councils, creating forums for Veterans to offer feedback on research topics, and hosting town hall sessions that allow Veterans to provide feedback.

Our work extends prior research, which sought to identify VA priorities. One previous study convened real‐time “state‐of‐the‐art” conferences, which included representatives from VA operational offices and discussed topics identified through a literature review [19]. Another study convened a Delphi panel of researchers and operational experts, with findings shared back with Veteran groups [20]. A different study used qualitative methods and a national survey to engage VHA leaders in nominating and rank‐ordering priorities to inform the prioritization of quality improvement investments [21]. Our approach similarly sought to identify VA priorities, but we designed our evaluation to directly compare priorities identified by VA leaders and those found to be important for Veterans. Several priorities identified in prior studies were consistent with this evaluation, including improving the measurement of access, ensuring that subpopulations receive access, and enhancing communication and information sharing with Veterans. However, we also found that Veterans and VA leaders expressed a need to prioritize health benefits and workforce concerns in the future, two areas not previously represented in the literature.

The approach we used to identify priority areas has several strengths, including the inclusion of multiple interested groups, a systematic approach to data collection that ensured a high participation rate, and the use of rigorous methods for qualitative data analysis. However, there are several limitations. For the document review, the sources and structure of the documents varied widely, making it difficult to carry out a formal, systematic review. We opted for a more pragmatic approach, focusing on the use of document content analysis to identify priority areas and recommendations. For focus groups and interviews, it was not feasible to record sessions and obtain transcripts. To overcome this challenge, we used well‐established rapid qualitative analysis techniques to ensure comprehensive data collection. We approached qualitative coding with a focus on specific areas for improvement in the future, but alternative approaches using broader groupings could be helpful in other contexts. It is also important to note that our findings are particularly relevant for strategic planning work within the VA and similar integrated health systems, but may not generalize readily to other settings. The focus of this evaluation was on broad priority areas relevant across multiple segments of VA, meaning that priorities within specific topic areas (e.g., VA research priorities) would be best addressed through additional research and evaluation projects. Lastly, our findings reflect priorities as identified in 2024 and may not be consistent with those that would be identified if data collection were to be repeated. There is a need to conduct further evaluations to understand how priorities may evolve in response to organizational changes.

## 6 Conclusions

We used a range of qualitative data collection methods and worked with multiple interested groups to identify VA priorities and recommendations for improvement. Despite differences across the data sources, our findings suggest important shared priorities to emphasize as VA looks to the future: improving access to care, delivering efficient and effective processing of benefits, ensuring the needs of special Veteran groups are met, and sustaining an adequate workforce to care for Veterans. Additional work should focus on achieving an equitable approach to investing limited organizational resources. Other health systems could also emulate our rapid evaluation approach in resource‐ and time‐limited settings where the perspectives of multiple key groups need to be integrated.

## Funding

This work was supported by the VA Quality Enhancement Research Initiative (EBP 22‐106) and VA Health Systems Research (CIN 13‐408).

## Disclosure

This manuscript represents the views of the authors and does not represent the views of the Department of Veterans Affairs or the US government. J.P.D. receives personal fees from the American College of Emergency Physicians as a methodology/statistics editor for *Annals of Emergency Medicine*.

## Supporting information


**Appendix S1:** Inclusion criteria for document analysis.
**Appendix S2:** Keywords for database searches.
**Appendix S3:** Guides for focus groups and semi‐structured interviews.
**Appendix S4:** Codebook for priority areas.
**Appendix S5:** Documents identified and included in document analysis by source.
**Appendix S6:** Frequency counts for all priority areas identified from document analysis.

## Data Availability

Research data are not shared.
