# Supplementary material for: Identifying Priorities for the Department of Veterans Affairs Strategic Plan: A Rapid Multi‐Method Evaluation
Source: Learn Health Syst. 2026 Feb 12;10(2):e70068. doi: 10.1002/lrh2.70068 (PMC12900616; doi:10.1002/lrh2.70068)
Supplement: Supplementary file 1 — Appendix S1: Inclusion criteria for document analysis. Appendix S2: Keywords for database searches. Appendix S3: Guides for focus groups and semi‐structured interviews. Appendix S4: Codebook for priority areas. Appendix S5: Documents identified and included in document analysis by source. Appendix S6: Frequency counts for all priority areas identified from document analysis. [file LRH2-10-e70068-s001.docx]

### APPENDICES

### Supplemental Methods:

### Appendix 1: Inclusion criteria for document analysis

### Appendix 2: Keywords for database searches

### Appendix 3: Guides for focus groups and semi-Structured interviews

### Appendix 4: Codebook for priority areas

### Supplemental Results:

### Appendix 5: Documents identified and included in document analysis by source

### Appendix 6: Frequency counts for all priority areas identified from document analysis

### APPENDIX 1

### Inclusion criteria for document analysis

| **Source** | **Inclusion Criteria** | **Exclusion Criteria** |
| --- | --- | --- |
| **Academic/Research Databases** | Targeted search terms in article title **(Appendix 2)** | Grey literature (abstracts, conference presentations), book chapters, disease- specific, program-specific |
| **Government Websites** | VA-specific reports, testimonials |  |
| **Nationally Syndicated**  **Newspaper Websites** | VA-specific, US-focus,  available in repositories | State-specific, opinion  pieces, commentaries, letters |
| **Research Organizations** | VA-specific press releases, legislative papers, manuscripts |  |
| **VA Staff Groups** | Press releases |  |
| **Veteran Advocacy and Assistance Groups** | Press releases, legislative paper |  |

## **APPENDIX 2**

## **Keywords for database searches**

| **PubMed**  **(and select non-indexed journals)** | **JSTOR**  **(and select non-indexed journals)** |
| --- | --- |
| ("veteran"[Title] AND "benefits"[Title]) OR  ("veteran"[Title] AND "future"[Title]) OR  ("veteran"[Title] AND "priority"[Title]) OR  ("veteran"[Title] AND "priorities"[Title]) OR  ("veteran"[Title] AND "need"[Title]) OR  ("veteran"[Title] AND "needs"[Title]) OR  ("veteran"[Title] AND "improve"[Title]) OR  ("veteran"[Title] AND "improvement”[Title]) OR  ("veteran"[Title] AND "concern"[Title]) OR  ("veteran"[Title] AND "concerns”[Title]) OR  ("veteran"[Title] AND "problem"[Title]) OR  ("veteran"[Title] AND "problems"[Title]) OR  ("veteran"[Title] AND “opportunity”[Title]) OR  ("veteran"[Title] AND “opportunities”[Title]) OR  ("veteran"[Title] AND “demographics”[Title]) OR  ("veteran"[Title] AND “fix”[Title]) OR  ("veteran"[Title] AND "resources"[Title]) OR  ("veteran"[Title] AND "services"[Title]) OR  ("veteran"[Title] AND “success”[Title]) OR  ("veteran"[Title] AND “wins”[Title]) | ((ti:"veteran") |

**APPENDIX 3**

**Guides for focus groups and semi-structured interviews**

1. **VEP focus group guide and questions**

[All CEIR staff arrive with cameras on]

Hello! Thank you for joining!

I am [name]. I am a VA employee from the VA QUERI Center for Evaluation and Implementation Resources, also known as CEIR. CEIR provides evaluation support to different VA offices. I am joined today by two of my colleagues, [moderator introduces notetakers]. They are working on this project and will be taking notes of our discussion.

Before we begin, I need to read a brief introduction to this project. It is a little lengthy, but please bear with us.

You have been asked here today because of your involvement on your Veteran Engagement Panel, sometimes known as a Veteran Engagement Council. For the next hour, you will be part of a focus group. In a focus group, people come together to talk about a specific topic and give their ideas.

As the moderator, I get to ask the questions, but I am not an expert on the topic. I am simply guiding the discussion today.

The purpose of today’s focus group is to get your feedback about Veteran needs, issues that are important to you as a Veteran, things that you think VA does well and should continue, and things that you think VA could improve. We are asking for your feedback on these topics because VA is developing its next Strategic Plan and wants to give Veterans a voice in the creation of this Plan.

We are interested in your feedback regarding VA benefits and services, such as disability compensation, pension, education and training, health care, home loans, insurance, employment, and burial. You are certainly welcome to share your experience with VA healthcare, but we also want to get your feedback on other benefits and services that VA provides.

Do you have any questions about anything I just said? [answer questions]

This is not a research study, but an internal VA quality improvement project. When finished, we will compile the notes we collect and write a report for the VA Office of Enterprise Integration (OEI). This is the VA office responsible for leading the Strategic Planning process.

Your participation in this focus group is completely voluntary. We will not use your name or anything that identifies you in any reporting of the focus group discussions. However, we cannot guarantee that what you say will not be repeated by someone else in this focus group. We are not asking you to share any personal or sensitive information and you do not have to speak up and say anything if you do not want to. You can choose not to respond to any question you do not want to answer.

We do have a few requests of you.

- Only one person speaks at a time. This is important as our goal is to take accurate notes. It is difficult to write down feedback if there are multiple voices at once.
- Please remain on mute, if possible, to minimize background noise and interruptions while others are speaking.
- If you are comfortable and able to do so, we’d appreciate having cameras on during our conversation.
- Everyone should have a chance to speak if they want. You are also welcome to use the chat.
- We recognize that thoughts about the future and direction of VA can be influenced by individual life experiences, beliefs, and political opinions, however, we do not want to focus on politics today.
- Please be respectful of your fellow Veterans’ views. We recognize that Veterans have different opinions about VA.

We are most interested to hear about your personal experiences, opinions, and views on what we discuss, so please provide open and honest feedback. There are no right or wrong answers to our questions. It is okay to reflect on both positive and negative experiences. Your views are extremely valuable to us, and we are here to learn from you.

That is the end of our introduction. Now that we have told you more about the focus group, we would like to make sure that everyone still wants to participate. We would like to have everyone go around and state their first or preferred name, their branch of military service, and whether they agree to participate.

[wait for participants to give their verbal consent]

Thank you. Let’s get started. The notetakers for this project are going to turn off their cameras so that they can focus on taking notes.

[note-takers turn off cameras]

[Questions highlighted in yellow are most important]

1. (Needs) What do you think are the greatest unmet needs that you or your fellow Veterans face today?
   - Probe: needs of new Veterans;
   - Probe: needs of Veterans who are eligible for VA services but do not take advantage of them
2. (Fixes) What are a few things VA could change to meet these needs?
   - Probe: What specific improvements would need to be in place for changes to occur?
   - Probe: What do you think would help VA to make these changes?
3. (Strengths) What VA benefits, services, or resources does VA need to continue or expand in the future?
4. (Waste) What benefits, services, or resources that VA provides should have a lower level of priority, given the needs of Veterans?
5. (Opportunities) In what areas do you think VA needs to improve in the next 5 to 10 years?
6. (Growth) Do you have any unique, bold, or out-of-the-box ideas to make VA better?

[If time permits]

We are nearing the end of our time.

1. Is there anything else that you think needs to be communicated to VA leadership? If so, what?

We have come to the end of our questions. Before we wrap up, we’d like to get an idea of your generation, or age grouping, to better understand the background of our population for each focus group. If you are comfortable sharing, please let me which of the following generations you fall into. I’ll read a brief list. Either raise your hand or say “that’s me.”

[do not read ages unless asked]

- The Post-War Generation, that is, those born between 1928-1945 (79 – 96)
- Baby Boomers, that is, those born between 1946-1964 (60-78)
- Generation X, that is, those born between 1965-1980 (44 – 59)
- Millennials, that is, those born between 1981-1996 (28 – 43)
- Generation Z, that is, those born between 1997-2012 (27 and under)

We would like to thank you all for your active participation in today’s discussion.

1. **EBPS interview guide and questions**

1:1 Interview Version

[All CEIR staff arrive with cameras on]

Hello! Thank you for joining!

I am [name]. I am a VA employee from the QUERI Center for Evaluation and Implementation Resources, also known as CEIR. I am joined today by two of my colleagues, [interviewer introduces notetakers]. They are working on this project and will be taking notes of our discussion.

Before we begin, I need to read a brief introduction to this project. It is a little lengthy, but please bear with us.

You have been asked to participate in an interview because of your office’s involvement with the VHA Evidence-based Policy Subcommittee, or EBPS. Given the importance of the VA Strategic Plan to the EBPS’s work, we would like to get your perspectives related to the needs of Veterans and VA, things that you think VA does well and should continue, things that you think VA could improve, and suggested priority areas for the next iteration of the VA Strategic Plan.

We are interested in your feedback on VA healthcare, as well as broader VA benefits and services, such as disability compensation, pension, education and training, health care, home loans, insurance, employment, and burial.

Do you have any questions about anything I just said? [answer questions]

This is not a research study, but an internal VA quality improvement project. When finished, we will compile the notes we collect and write a report for the VA Office of Enterprise Integration (OEI), which is leading the Strategic Planning process.

Your participation in this interview is completely voluntary. We will not use your name or anything that identifies you in any reporting of our interviews. You can choose not to respond to any question you do not want to answer.

If you are comfortable and able to do so, we would appreciate having cameras on during our conversation. You are also welcome to use the chat feature within Zoom to share additional information.

That is the end of our introduction. Now that we have told you more about the interview, we would like to make sure that you still want to participate.

[wait for participant to give their verbal consent]

Thank you. Let’s get started. The notetakers for this project are going to turn off their cameras so that they can focus on taking notes. [note-takers turn off cameras]

Group Interview Version:

[All CEIR staff arrive with cameras on]

Hello! Thank you for joining!

I am [name]. I am a VA employee from the QUERI Center for Evaluation and Implementation Resources, also known as CEIR. I am joined today by two of my colleagues, [interviewer introduces notetakers]. They are working on this project and will be taking notes of our discussion.

Before we begin, I need to read a brief introduction to this project. It is a little lengthy, but please bear with us.

You have been asked to participate in an interview because of your office’s involvement with the VHA Evidence-based Policy Subcommittee, or EBPS. Given the importance of the VA Strategic Plan to the EBPS’s work, we would like to get your perspectives related to the needs of Veterans and VA, things that you think VA does well and should continue, things that you think VA could improve, and suggested priority areas for the next iteration of the VA Strategic Plan.

We are interested in your feedback on VA healthcare, as well as broader VA benefits and services, such as disability compensation, pension, education and training, health care, home loans, insurance, employment, and burial.

Do you have any questions about anything I just said? [answer questions]

This is not a research study, but an internal VA quality improvement project. When finished, we will compile the notes we collect and write a report for the VA Office of Enterprise Integration (OEI), which is leading the Strategic Planning process.

Your participation in this interview is completely voluntary. We will not use your name or anything that identifies you in any reporting of our interviews. However, we cannot guarantee that what you say will not be repeated by someone else in this group.

We are not asking you to share any personal or sensitive information. You can choose not to respond to any question you do not want to answer.

We do have a few requests of you.

- Only one person speaks at a time. This is important as our goal is to take accurate notes. It is difficult to write down feedback if there are multiple voices at once.
- Please remain on mute, if possible, to minimize background noise and interruptions while others are speaking.
- If you are comfortable and able to do so, we’d appreciate having cameras on during our conversation.
- You are welcome to use the chat.

Your views are extremely valuable to us, and we are here to learn from you.

That is the end of our introduction. Now that we have told you more about the interview, we would like to make sure that everyone still wants to participate. We would like to have everyone go around and state their first or preferred name and whether they agree to participate.

[wait for participants to give their verbal consent]

Thank you. Let’s get started. The notetakers for this project are going to turn off their cameras so that they can focus on taking notes.

[note-takers turn off cameras]

Interview Questions:

1. *What issues will challenge the Department of Veterans Affairs within the next 5 to 10 years?
2. *In what areas do you think VA needs to improve in the next 5 to 10 years?
3. What VA benefits, services, or resources should VA expand in the future?
4. What benefits, services, or resources that VA provides should have a lower level of priority, given the needs of Veterans?
5. Tell me your most unique, bold, or out-of-the-box idea to make VA better.
6. We have been talking about the VA as an agency for a while. Let’s now zoom in on Veterans. What challenges do you foresee for Veterans, particularly at-risk Veterans, over the next 5 to 10 years?
   - Probe: needs of new Veterans
   - Probe: needs of Veterans who are eligible for VA services but do not take advantage of them
   - Probe: What specific improvements would need to be in place for changes to occur?
   - Probe: What do you think would help VA to make these changes?
7. *Now I would like to narrow in and ask specifically about the Strategic Plan. What are some important areas you believe should be included in VA’s next Strategic Plan?
8. Is there anything else that you think needs to be communicated to the Office of Enterprise Integration regarding the development of the next VA Strategic Plan? If so, what?
9. *Lastly, are there other people you think could provide valuable insight on this process that we should interview? If so, what are their names and program offices?

We would like to thank you all for your active participation in today’s discussion.

1. **Snowball sampling key informant (non-EBPS) interview guide and questions**

[Interviewer should review tracking spreadsheet in advance to know which EBPS member recommended the participant]

[All CEIR staff arrive with cameras on]

Hello! Thank you for joining!

I am [name]. I am a VA employee from the QUERI Center for Evaluation and Implementation Resources, also known as CEIR. I am joined today by two of my colleagues, [interviewer introduces notetakers]. They are working on this project and will be taking notes of our discussion.

Before we begin, I need to read a brief introduction to this project. It is a little lengthy, but please bear with us.

You have been asked to participate in this interview at the recommendation of a member of the VHA Evidence-based Policy Subcommittee. We would like to get your perspectives related to the needs of Veterans and VA, things that you think VA does well and should continue, things that you think VA could improve, and suggested priority areas to inform the next iteration of the VA Strategic Plan.

We are interested in your feedback on VA healthcare, as well as broader VA benefits and services, such as disability compensation, pension, education and training, health care, home loans, insurance, employment, and burial.

Do you have any questions about anything I just said? [answer questions]

This is not a research study, but an internal VA quality improvement project. When finished, we will compile the notes we collect and write a report for the VA Office of Enterprise Integration (OEI), which is leading the Strategic Planning process.

Your participation in this interview is completely voluntary. We will not use your name or anything that identifies you in any reporting of our interviews. You can choose not to respond to any question you do not want to answer.

If you are comfortable and able to do so, we would appreciate having cameras on during our conversation. You are also welcome to use the chat feature within Zoom to share additional information.

That is the end of our introduction. Now that we have told you more about the interview, we would like to make sure that you still want to participate.

[wait for participant to give their verbal consent]

Thank you. Let’s get started. The notetakers for this project are going to turn off their cameras so that they can focus on taking notes. [note-takers turn off cameras]

Interview Questions:

1. *What issues will challenge the Department of Veterans Affairs within the next 5 to 10 years?
2. *In what areas do you think VA needs to improve in the next 5 to 10 years?
3. What VA benefits, services, or resources should VA expand in the future?
4. What benefits, services, or resources that VA provides should have a lower level of priority, given the needs of Veterans?
5. Tell me your most unique, bold, or out-of-the-box idea to make VA better.
6. We have been talking about the VA as an agency for a while. Let’s now zoom in on Veterans. What challenges do you foresee for Veterans, particularly at-risk Veterans, over the next 5 to 10 years?
   - Probe: needs of new Veterans
   - Probe: needs of Veterans who are eligible for VA services but do not take advantage of them
   - Probe: What specific improvements would need to be in place for changes to occur?
   - Probe: What do you think would help VA to make these changes?
7. *Now I would like to narrow in and ask specifically about the Strategic Plan. What are some important areas you believe should be included in VA’s next Strategic Plan?
8. Is there anything else that you think needs to be communicated to the Office of Enterprise Integration regarding the development of the next VA Strategic Plan? If so, what?

We would like to thank you all for your active participation in today’s discussion.

**APPENDIX 4**

**Codebook for priority areas**

***Health Benefits:*** *eligibility, need to expand*

***Conditions and Treatments:*** *mental health, polytrauma, substance abuse, infertility*

***Military Exposure****: toxic exposure*

***Homeless Veterans:*** *needs identified*

***Connected Care:*** *telehealth, virtual care*

***Suicide/Suicide Prevention:*** *resources and services specific to suicide and suicide prevention, needs identified*

***PTS/PTSD:*** *resources and services specific to PTS/PTSD, needs identified*

***Women’s Health:*** *resources and services specific to women Veterans, needs identified*

***Wellness Programs:*** *MyHealthyVet, nutrition, quitting smoking, vaccines and immunizations, prevention/wellness, weight management, whole health*

***Special Groups:*** *combat Veterans, returning service members, rural Veterans, seniors and aging Veterans, volunteers, peer support, patient advocates, traumatic brain injury, Veteran parents, reserves*

***Caregivers and Families:*** *resources and services specific to caregivers*

***Community Care and Privatization:*** *replacement and/or supplementation of services with private-sector care*

***Access and Continuity of Care:*** *wait times, technology, service availability, communication between providers*

***Infrastructure:*** *aging facilities*

***Patient Safety:*** *adverse events, prevention, and reduction of unnecessary events*

***Outreach, Transition and Economic Development:***  *transition to civilian life, connection to local resources (e.g., non-profit), integration between DoD and VA*

***Compensation:*** *disability claims, processing times*

***Disability Claims Digital Experience:*** *backlog*

***Education, Training, Employment:*** *predatory claims, support for scholarships, student housing allowance, childcare stipend, employment counseling, GI bill*

***Housing and Home Loans:*** *housing support, HUD-VASH*

***Life Insurance and Pension:*** *support for Veterans and survivors*

***Applying for Benefits:*** *need for modernization, DBQs*

***Transportation Services:*** *travel solutions and options for reimbursement*

***Burial and Memorial Services:*** *burial allowance, increase dependency and indemnity benefits for survivors*

***Long-Term Care:*** *nursing homes, assisted living*

***Customer Relations:*** *communication, trust, transparency, accountability, stigma*

***Veteran Belonging:*** *concordance between Veteran and provider, Veteran experience*

***Social Factors/Support/Needs:*** *role of social networks and needs*

***Legislation:*** *descriptions of what legislation is intended to do****;*** *VA MISSION Act, Choice Act, National Defense Authorization Act, PACT Act (co-code as appropriate)*

***Food Insecurity:*** *impacts to health, need for additional benefits and services (co-code as appropriate)*

***Budget and Funding:*** *impacts of VA and federal budget on benefits and services (co-code as appropriate)*

***Data Management:*** *systems (e.g.,* *HEDIS, VistA, EHR), privacy and cybersecurity, data integration, lack of data on subgroups*

***Workforce:*** *staffing and retention, leadership, rural needs, medically underserved*

*Rural Workforce:*

***Parking Lot:*** *Incarceration, medicalization of marijuana, shifts in demographics, coordination w DoD, acquisition management, CBOCs*

**APPENDIX 5**

**Documents identified and included in document analysis by source**

| **Document Source** | **Identified*** | **Included**** |
| --- | --- | --- |
| **Academic/Research Databases** | **134 (45.3%)** | **53 (31.2%)** |
| PubMed | 52 | 29 |
| JSTOR | 46 | 14 |
| Non-Indexed | 36 | 10 |
| **Government** | **46 (15.5%)** | **44 (25.9%)** |
| Government Accountability Office *(GAO)* | 46 | 44 |
| **Nationally Syndicated Newspaper Websites with Searchable Databases** | **49 (16.6%)** | **33 (19.4%)** |
| New York Times *(NYT)* | 14 | 12 |
| Washington Post *(WP)* | 10 | 4 |
| Wall Street Journal *(WSJ)* | 25 | 17 |
| **Veterans Advocacy and Assistance Groups** | **31 (10.5%)** | **21 (12.4%)** |
| American Legion | 8 | 8 |
| American Veterans *(AMVETS)* | 1 | 1 |
| Disabled American Veterans *(DAV)* | 5 | 3 |
| Paralyzed Veterans of America *(PVA)* | 7 | 5 |
| Veterans of Foreign Wars *(VFW)* | 2 | 2 |
| Vietnam Veterans of America *(VVA)* | 7 | 1 |
| Wounded Warrior Project *(WWP)* | 1 | 1 |
| **Research Organizations** | **22 (7.4%)** | **12 (7.1%)** |
| RAND Corporation | 11 | 6 |
| Pew Research Center *(PEW)* | 2 | 1 |
| Center for American Progress *(CAP)* | 9 | 5 |
| **VA Staff** | **14 (4.7%)** | **7 (4.1%)** |
| American Federation of Government Employees *(AFGE)* | 14 | 7 |
| **Total** | **296** | **170** |
| Note: * percent of identified documents from this source.  ** percent of included documents from this source. | | |

APPENDIX 6

Frequency counts for all priority areas identified from document analysis

 ^*^Miscellaneous and Legislation not included in frequency counts
